# An investigation into the potential effects of infrapopulation structure and other sources of sampling error, on population genetic studies of the transmission of *Schistosoma japonicum* (Trematoda: Digenea)

**DOI:** 10.1186/s13071-016-1454-0

**Published:** 2016-03-21

**Authors:** Guan-Nan Huo, Liang Liu, Hong-Bin He, Stephen W. Attwood

**Affiliations:** State Key Laboratory of Biotherapy, West China Hospital, West China Medical School, Sichuan University, Chengdu, People’s Republic of China; Hunan Institute of Parasitic Diseases, Yueyang, Hunan People’s Republic of China; Department of Life Sciences, The Natural History Museum, London, UK

**Keywords:** *Schistosoma japonicum*, Microsatellite, Infrapopulation, Population genetics, Hardy-Weinberg, Sampling bias, Organotropic, Mating, Genetic differentiation

## Abstract

**Background:**

*Schistosoma japonicum* remains a major challenge to human and animal health. Earlier microsatellite-based studies reported possible definitive-host-specific private alleles within *S. japonicum*, opening the possibility that different definitive hosts might harbour different parasite strains. Previous investigations have also detected near-identical multilocus genotypes in populations of adult worms - possibly the result of mutations occurring during the asexual (intramolluscan) phase of clonal expansion. Research has also revealed extensive deviations from Hardy-Weinberg Proportions (HWP) and conflicting results among studies. The present study was performed to examine some of the potential effects of infrapopulation structure on microsatellite-based studies of the transmission ecology of *S. japonicum*. Potential sources of bias considered included organotropic distribution of worms, non-random mating and corrections for clonal expansion.

**Results:**

Stool samples from naturally infected hosts were used to infect snails in the laboratory and thereby expose mice. 274 individual worms were typed at seven microsatellite loci. Removal of individuals bearing duplicate MLGs (as a correction for presumed clonal expansion) had an impact on both HWP and organotropic genetic differentiation. The study found no evidence that heterozygote deficiencies were caused by a Wahlund effect. Female-male pairings appeared to be random and there was no evidence for mate choice by heterozygosity. There was some indication that excess heterozygosity, induced by clonal expansion, can offset heterozygote deficiencies caused by small population size or populations fragmented by parasite control efforts.

**Conclusions:**

The view is supported that miracidia are preferable to adult worms in investigations into host-specific parasite lineages. Where adults must be used, extreme care should be taken with regard to sampling if infrapopulations of small animals are compared with those of larger animals; this is because of organotropic patterns in genetic variation and the tendency to sample from different organs in differently sized hosts. As corrections for clones may accentuate signals of population subdivision, corrections should only be made if tests for clonal expansion prove positive. Finally, evidence for heterozygote deficiency caused by small sample size, calls for carefully designed random and comprehensive sampling strategies for *S. japonicum* in China, where control efforts have greatly fragmented parasite populations.

**Electronic supplementary material:**

The online version of this article (doi:10.1186/s13071-016-1454-0) contains supplementary material, which is available to authorized users.

## Background

### Population genetic studies of schistosomiasis transmission in China

Schistosomiasis is a parasitic disease transmitted by certain freshwater snail species (intermediate hosts); the disease is the result of infection by species of *Schistosoma* (Trematoda: Digenea). At least 261 million people required preventive treatment for schistosomiasis in 2013 and the disease can cause serious and debilitating illness [1]. In China schistosomiasis is caused by *Schistosoma japonicum* and is transmitted by snail intermediate hosts belonging to the *Oncomelania hupensis* (Caenogastropoda: Pomatiopsidae) sub-species complex. People become infected when contacting water in which these snails live and into which they release a mobile and penetrative larval stage, the cercaria. The snails become infected by free-swimming miracidia which hatch from eggs passed by infected definitive hosts.

In spite of 50 years of snail and disease control *Schistosoma japonicum* still infects approximately one million people and 100,000 cattle in China [2]. Surveys performed between 2005 and 2008 on initially infection-free villages in Sichuan Province showed re-emergence in 61 % of the villages surveyed and overall prevalences of up to 43 % in humans and 65 % in cattle [3]. Across China over 360 thousand people are thought to be infected [4] and a further 65 million at risk of infection [5]. The area of habitat suitable for the snail intermediate hosts across China remained constant between 2008 and 2013 at approximately 1.08 million km^2^ [6]. In addition, the region of China suitable for *Oncomelania* is expected to show a northwards expansion of over 783,883 km^2^ by 2050, presumably due to global warming [7]. Clearly, schistosomiasis eradication is difficult and it is vital that we have adequate methods to combat the spread of infection. *Schistosoma japonicum* causes a true zoonosis, utilising a range of mammals as definitive host (including humans). In view of this a number of population genetic studies have been performed in order to understand definitive host use in schistosomiasis transmission in China and the Philippines, especially to determine which definitive host species are most important in maintaining transmission in human populations. Whilst these investigations have produced interesting findings, they also yielded unexpected results that were difficult to explain or showed inconsistency between studies. Consequently, the present study was performed in order to evaluate infrapopulation structure as a potential source of bias in population genetic analyses of schistosomiasis transmission.

Detailed population genetic studies of *S. japonicum* were not possible until the development of 8 polymorphic microsatellite markers suitable for fine-scale studies of *S. japonicum* [8]. The subsequent microsatellite based studies of *S. japonicum* from China yielded interesting results and represent remarkable achievements in the study of the process of transmission in nature (rather than simply in the laboratory). For example, a clustering of alleles from worms sampled in Sichuan and Yunnan (highland areas), relative to those of lowland areas, was detected using F_ST_ values and UPGMA; this was attributed to differences in definitive host use between regions [9]. In support of this Wang et al. [10] showed a clustering of worms from humans and bovines relative to those from goats, dogs, cats and pigs. The authors noted that their observation could be explained as an effect of parasite strain substructuring leading to differential transmission among definitive host species. In contrast Rudge et al. [11] reported a clustering of *S. japonicum* sampled from dogs and bovines in marshland areas and humans rodents and dogs in highland areas, but often found little differentiation among parasite sub-populations of different host types in sympatry; these authors suggested that patterns may differ even among local villages or between years. Rudge et al. [12] also showed how genetic structuring of Philippine *S. japonicum,* between humans and other hosts, differs significantly from that in marshland China. In addition, private alleles were detected between mouse and rabbit infrapopulations (adult worms) arising from exposure to the same sample of field derived cercariae, and multilocus genotypes (MLGs) of individual worms clustered by definitive host type in UPGMA; this is suggestive of host-induced selection [13]. More recently, it was observed that in many samples of adult worms the number of MLGs was much greater than the number of miracidia founding the sample; these near identical niMLGs were observed in cercariae and adult worms that had developed from clonally derived sibling cercariae. The niMLGs were assumed to result from somatic mutation during clonal reproduction at the sporocyst stage. The niMLGs were mostly sex linked but not linked to either sex chromosome [14]. The aforementioned exemplary studies mostly focused on the difficult task of performing natural experiments, rather than evaluating sampling procedures. Consequently, the present study was performed to look into the sampling process and potential sources of bias that can be accounted for in future studies.

### Handling niMLGs and the potential problems posed by infrapopulation structure

The observation of niMLGs appears to be a feature of most microsatellite based studies of *S. japonicum* transmission. In a study of the infection process using sibship analyses, and samples of cercariae and miracidia, many cercarial genotypes within single snails were found to differ in by one locus only; this is again suggestive of somatic mutation within the intramolluscan stage of the parasite [15]. Similar niMLGs have been detected in studies of *S. japonicum* using microsatellites to investigate geographical population genetic structure among Chinese provinces and other countries where the parasite occurs [16], as well as in research demonstrating bottlenecking in parasite populations by host type and through laboratory cycling [13]. The niMLGs were observed at the level of cercariae [15] and adult worms [13, 14, 16]. Different authors have used different methods to accommodate these niMLGs in *S. japonicum* studies; these range from including them in the analysis [13, 15], to deleting all but one representative of niMLG allele clusters identified by principal-coordinates analyses (PcoAs) [16, 17]. Neither of these approaches is ideal, as both have the potential to bias downstream analyses. The reduction in sample size through removing individuals identified as clones could inflate F_ST_ between populations by random loss of MLGs from a sub-sample of the data (i.e, a host-type or ecovar). Polymorphic loci such as microsatellites have high sampling variances when sample size is small [18]. In addition, for small populations (as one might encounter with *S. japonicum*), the sampling of gametes and fertilization to create zygotes causes random error in allele frequencies; this results in a deviation from Hardy-Weinberg Proportions (HWP), which becomes larger at small sample sizes. On the other hand, retaining clones within the analyses may lead to an excess of heterozygotes relative to random mating [18]. Clonal reproduction also effects an increase in allelic diversity, but a decrease in genotypic diversity, relative to a randomly mating population [19]. Clonality also has an impact on Linkage Disequilibrium (LD), generating non-random associations between loci [20] and a recent increase in the prevalence of particular clones will inflate LD [21]. An alternative approach, which is explored in this study, is to identify potential clones, assign clone-specific MLGs and then reassign the clones back to their original populations; thus eliminating spurious genetic variation within clone classes, but keeping all members of the original clones so that the distribution of MLGs among sub-populations in maintained. Clone detection approaches have been used in past studies (e.g., GENCLONE [22]) [14]; however, the clones were deleted and frequencies in the original distribution of MLGs among populations were lost. As the effects of niMLGs and their removal are uncertain, the present investigation is the first microsatellite based study to perform analyses with and without apparent clonal individuals.

The results of earlier studies (e.g., [10–12]) based on miracidia imply that definitive host-induced selection may be occurring; however, some of these observations can also be explained in terms of differences in parasite maturation rate. It is known that eggs coming from different hosts show different hatching rates (Mao [23]; Li et al. [24]). Differences in niche between strains could also cause problems in studies based on adult worms; the niche occupied by adults of even sister *Schistosoma* spp. can differ [25]. Similarly, it is well established that schistosomes migrate from the liver to the mesentery as they mature, and that this process occurs at different rates in different host taxa [26]. These phenomena can lead to loss of private alleles between life-cycle stages or infrapopulations because they create differences in the probability of recovering all worms, or of sampling all miracidia, between stages or host groups. In some definitive host groups adult worms (in the mesentery) are more readily recovered than immature worms in the liver, and worms mature at different rates in a rabbit and in a mouse (*ceteris paribus*). For example, such differences in sampling coverage could lead to observed genetic substructuring attributed to differential host-parasite lineages and generating apparent selection (or inconsistency in the findings of different authors) where adult worms are used; thus obscuring true patterns. Genetic studies of *Schistosoma* spp. typically recover only 12 % of the cercarial exposure and previous researchers [13] noted that alleles might be lost if sample sizes are too small. The same phenomenon may also explain the odd sex-specific (autosomal) MLGs observed by Yin et al. [14] if male and female worms migrate differently. Demonstration of potential sampling bias due to infrapopulation sub-structure would also further the case for the use of larval stages, such as the miracidia used in some earlier studies of definitive host-specific lineages [10–12], rather than adult worms.

### Apparent deviations from assumptions of Hardy-Weinberg Equilibrium

Departures from HWP have been a common feature of past microsatellite studies of *S. japonicum*. Significant heterozygote deficiencies, across all or most loci sampled, have been reported for miracidia [10, 12], cercariae [10] and adult worms obtained from laboratory infections of rabbits exposed to cercariae shed by field collected snails [9]. Heterozygote excesses have also been reported from rodents in hilly areas by one study of miracidia [11]. Some studies have simply reported deviations from HWP, whilst others have offered explanations for the deviations. Deficiencies in pig infrapopulations have been explained as an effect of bottlenecking due to pigs being tethered and exposed to a small parasite gene-pool or their exposure being restricted to a brief period of free-roaming as piglets [12]. Heterozygosity loss in bovines has been considered as due to their large size, which leads to a more structured infrapopulation when compared to smaller rodents that showed less deficiency [11], although, in other studies, rodents showed the greatest loss of heterozygosity [12]. Deficiencies across loci have been attributed to inbreeding, non-random mating or population sub-division (the Wahlund effect) [9, 16]. In addition to deviations from HWP, studies of adult worms have found many loci to be in genotypic linkage disequilibrium; this was assumed to be a result of inbreeding and nonrandom mating, partly due to population subdivision [9].

The impact of departures from HWP of course depends on the nature of the downstream analyses, and in many of the studies mentioned above HWP was not an important assumption. Nevertheless, some studies did employ the program STRUCTURE [27] to detect clustering of individuals into separate populations by region or by host (e.g., [11, 16]). As STRUCTURE assumes both linkage equilibrium and HWP when it estimates population structure, an alternative approach that made no assumptions about population genetic processes may have been better. Although in one study [11], the STRUCTURE analysis was repeated using only one (randomly selected) miracidium per host group in order to overcome the possible departures from HWE; this solution involves a loss of data and loci may still be linked. In addition, there are choices to be made regarding genetic differentiation metrics and confounding factors in these kinds of studies. Previous studies on *S. japonicum* used Wright’s F_ST_ [28], presumably generalised for loci with multiple alleles [29], and AMOVA (Analysis of Molecular Variance) to estimate parasite genetic differentiation among populations. The generalised F_ST_, when used on microsatellites showing high heterozygosity, may rarely exceed 0.25, making Wright’s [30] criterion, that a range from 0 to 0.05 indicates “little” genetic differentiation, inapplicable. In view of this, the proposed study was designed to make use of alternative distance measures that are more suitable for microsatellites and loci with many alleles. Although F_ST_ may remain the index of choice where migration is of interest [31], other metrics may be more useful in cases where genetic differentiation is the focus - such as (the standardised G_ST_) G’_ST_ [32] and Jost’s D [33], as the latter can be used to qualify estimates of diversity based on the former. D is also useful where the interest is in the allelic differentiation among populations [34]. AMOVA is also based on an estimator of Wright’s F_ST_ and the present data involved crossed factors, thus alternatives to AMOVA were sought.

### Aims of the present study

As no previous population genetic investigation into the organotropic distribution of *Schistosoma japonicum* within the definitive host had been performed; this study was undertaken to detect any clustering of MLGs, in different organs of the definitive host, that might affect studies into definitive-host induced selection or host-group specific parasite strains, that are based on adult worms. In addition, issues regarding HWE, methods used for detecting population structure, sex-specific patterns and methods of handling niMLGs are also considered, as these appear to be problematic or of interest in some earlier studies. Consequently, this study keeps track of the sampling locations of individuals in the definitive host, of the pairings of males and females and uses Discriminant Analysis of Principal Components (DAPC) to find clusters of MLGs within the data. DAPC makes no assumptions about population genetic processes such as HWE and linkage equilibrium. Unlike standard PCA, the DAPC is optimised to detect or to test for patterns or structure in the data. The study also uses a similarly assumption free procedure, implemented by the R-package allelematch, to detect clones and assign them to populations retaining the original population genetic structure. Allelematch is then used to detect and remove clones or to generate a new dataset with clones identified and retained, i.e. one that assumes niMLGs to be a consequence of mutations in the intramolluscan stage, with the “true” population comprising a smaller number of MLGs corresponding to genetically homogeneous clones. The analysis can then be performed using the original (Raw) data, the data with only a single copy of each clone retained (Filtered) and the data reconstructed with each individual assigned the MLGs of one clone (Clonal). The study may then compare the effects of using each data set. In addition, the study aimed to apply basic tests to determine or at least rule out potential causes for deviations from HWP, as these can shed light on the demographics or mating structure of the parasites, and to consider the use of alternative measures of population differentiation (to F_ST_).

The study also aimed to look at other factors which might affect patterns of population structure, such as sex, mating choice bias and potential effects of the laboratory host. Genetic differentiation between the sexes could also potentially bias studies because male worms tend to predominate at low levels of transmission. Cercariae from snails infected by a single female miracidium only, will produce a single-sex female infection of the definitive host, which is far less likely to establish than a male single-sex infection. Consequently, more males are likely to be sampled where transmission levels are low (e.g. in rodent populations for *S. japonicum*) than where they are higher (e.g. among bovines). Mating bias is also important, as females paired with males are more likely to survive in the host, and also more likely to be carried by the males to the mesenteric blood vessels; this would make paired females slightly more likely to be sampled in bovines than in rodents (unless special efforts were taken in sampling, as in the present study). The findings of the study were intended to assist in the design of experiments involving comparisons of *S. japonicum* in different definitive host groups, for example by demonstrating the benefits of studies based on larvae.

## Methods

### Parasite sampling

In May 2010, a sample of *Oncomelania hupensis* snails was collected from 19 villages of Matang Township, Yueyang County, Hunan Province, China. Autochthonous fecal samples were also collected from bovines. Eggs were recovered from the fecal samples and hatched. Snails were exposed to approximately three miracidia each. The exposures yielded 13 infected snails which shed cercariae; these were used to expose ten inbred female BALB/c mice to 50 cercariae each according to the following procedure. Snails were maintained in a dormant state on dry filter paper for two weeks before shedding. The snails were then placed individually in water and exposed to bright light for three hours. Cercariae that had emerged from each snail were then transferred to the moistened shaved abdomen of an anaesthetised mouse. Mice were anesthetised by intraperitoneal injection of sodium pentobarbital. The mice were exposed simultaneously (not in series) to cercariae from randomly selected snails, by stratified random sampling (each mouse was exposed to cercariae from each of the snails). The random selection of snails/cercariae continued until each mouse had been exposed to exactly 50 cercariae. The mice were sacrificed after 5 weeks, as this marks the onset of egg laying in *S. japonicum* [35] and animal suffering is minimised by this protocol. After sacrifice, the mice were dissected into citrate-saline and adult worms carefully removed into normal saline. The worms were extracted from the liver, hepatic-portal-vein (Hpv) and mesenteric blood vessels by a team of experienced technicians who periodically were randomly shuffled among mice to reduce effects of individual researcher technique. After manual removal of worms, the livers and mesentery were placed in normal saline in the dark at 4 °C, as this encouraged the emergence of any remaining worms. Final dissection of these organs was performed to maximise the likelihood that all worms had been recovered. The worms were fixed in 100 % ethanol immediately after removal. Worms *in copula* were fixed as such, so as to retain the natural pairing of males and females - although pairs of worms emerging at 4 °C would probably have separated; however, most worms so recovered were small males.

### Ethics statement

The exposures used in this study were part of a cycle of parasite maintenance established to provide material for immunological studies. Consequently, no animals were sacrificed specifically for the purpose of the present study, and it was not possible to sample the miracidia directly. Both studies were approved by the Animal Research Ethics Committee of Hunan Institute of Parasitic Diseases, Yueyang Hunan, and were in accordance with the guidelines of the Association for Assessment and Accreditation of Laboratory Animal Care International.

### Genetic sampling

Worms were removed from ethanol and rehydrated in TE buffer (pH 8.0), any paired worms were separated and the pairings noted, the sexes of single worms were also recorded. DNA was then extracted following the HotSHOT [36] extraction protocol. Nine microsatellite loci were amplified using published PCR primer sequences [37] (namely SjP23, SjP32, SjP37, SjP39, SjP42, SjP45, SjP54, SjP60, SjP88 – and referred to as LocusA to LocusH and LocusJ in this paper, respectively) and the M13 labelling PCR protocol [38]. The aforementioned PCR primers were chosen because these represented a set of tested [37] primers for highly polymorphic, pure tri-nucleotide repeat loci, sited in non-coding regions and with no evidence for null alleles or allelic drop out. The PCR products were run on a Applied Biosystems 3730xl DNA Analyzer. Raw data were analysed using GeneMapper4.0 (Applied Biosystems, Foster city, CA, USA), and product sizes were exported to a CSV format file. Tandem v1.09 [39] was then used to round allele sizes to multiples of tri-nucleotide repeats and thereby to bin alleles in the most consistent way relative to each other. The Tandem procedure revealed no outliers and that no individual had rounding errors above 0.75 for any locus.

Allelematch v2.5 [40] was used to identify, and to assign individuals to, any clones present in the population, as well as to detect and resolve any niMLGs assumed to be due to mutation during cercariogenesis. Allelematch detects similarities among individuals using a metric of the Hamming distance [41] and then employs hierarchical clustering and the dynamic method of the Dynamic Tree Cut package for R [42] to identify clusters on a resulting dendrogram. The approach was chosen because it does not involve any population genetic parameters (e.g. does not assume HWE). The output of allele match was used to produce two datasets, one with a single individual (MLG) representing each clone (Filtered) and one with all individuals included but each assigned to their respective clone (Clonal), in addition to the original (Raw) dataset. Allelematch considers the original distribution of individuals among sub-populations when assigning to clones. The analyses subsequently described were then performed on all three datasets in order to assess the impact of clonality (arising from rounds of clonal reproduction within the snail intermediate host as the cercariae were produced).

### Basic population genetics

The R package Pegas v0.8.1 [43] was used to obtain null allele frequency estimates based upon the forumulas of Brookfield [44], with 1000 bootstraps used to generate the distribution of the expected number of homozygotes for each allele at each locus based upon the observed allele frequencies at that locus. The R package Poppr v2.0.1 [45] was also used to calculate the Index of Association and Standardized Index of Association, with *P*-values from one-sided permutation tests (this gave values for r, in fact *r*¯*d* a less biased metric that allows for the number of loci sampled [46]). Tests for HWP were performed using the hw.test function of Pegas, with an exact test based on 5,000 Monte Carlo permutations of alleles. Other basic parameters used to describe the data (such as allelic richness and alleles per locus) were computed using the R package PopGenReport v2.2.1 [47]. The R package diveRsity v1.9.73 [48] was used to compute Weir and Cockerham’s estimation of F_ST_ (theta), G_ST_, G’_ST_, and Jost’s D with confidence intervals among populations obtained by pairwise bootstrap. Hill & Robertson’s [49] *r* was calculated using NeEstimator v2.01 [50].

### Examination of population structure

Principal Component Analysis (PCA) was used to detect any potential structure within the parasite infrapopulations, particularly with regard to organotropic distribution between the Liver, Hpv and Mesentery sub-populations. Discriminant Analysis of Principal Components (DAPC) was then used to test for the significance of any clustering detected by PCA.

Bartlett’s Test and the Fligner-Killeen test (native functions in R v3.2.2 [51]) were used to detect any significant differences among the variables in order to determine if scaling was required before PCA. The PCAs were performed using the dudi.pca (duality diagram PCA) function of the R package ade4 v1.7.2 [52]. Linear discriminant function analyses for population differences were undertaken using the principal components generated in the DUDI PCAs in ade4. The DAPCs were implemented using the R package adegenet v2.0.1 [53]. DAPC was chosen as a method to test for population structure because it does not rely on a particular population genetics model, and is thus free of assumptions about Hardy-Weinberg equilibrium or linkage disequilibrium [54]. DAPC provides membership probabilities of each individual for the different groups based on the retained discriminant functions; these can be interpreted as proximities of individuals to the different clusters in a similar way to the results from STRUCTURE. Membership probabilities also provide indications of how clear-cut genetic clusters are. Loose clusters will result in fairly flat distributions of membership probabilities of individuals across clusters, pointing to possible admixture [54]. The DAPCs were performed with the number of Principal Components (PCs) retained chosen to maximise the α-score (using the optim.a.score function of adegenet). The optimum number of PCs was also determined by cross-validation using the xvalDapc function. Cross-validation involves splitting the data into a training dataset and a validation dataset. The extent to which the analysis accurately predicts the group membership of excluded individuals (those in the validation set) is used to identify the optimal number of PCs to retain. At each level of PC retention, the sampling and Linear Discriminant Analysis (LDA) procedures were repeated 5,000 times. The number of discriminant functions to retain was determined by inspection of the graph of variance explained by the PCA. In addition to the group memberships function of DAPC, the find.cluster function of adegenet was also used; this involves running successive K-means, on PCA transformed data, with an increasing number of clusters (K). For each model, a BIC is computed as a statistical measure of goodness of fit. Test of the sum of a discriminant analysis eigenvalues (divided by the rank), that is for significance of the eigenvalues from the LDA, were performed using a non-parametric version of the Pillai’s test, as implemented by the randtest function (on the discrimin object) in adegenet, with 5000 replicates.

### Other sources of population structure: sex, mating and laboratory host

Pearson’s Chi-squared tests with simulated *P*-values (based on 5,000 replicates) were used to detect any significant difference among the mice or male and female worms in terms of organotropic distribution. Basic population genetic parameters were estimated for male and female worms alone, in order to detect, for example, sex-specific differences in deviations from HWP.

Mating individuals may be predominantly paired with genetically dissimilar mates, in order to increase the genetic variability of offspring [55–57], or genetically similar mates, in order to preserve the link between locally co-adapted genes [58, 59]. In addition, heterozygous mates maybe more commonly involved in pairings, as the most heterozygous mates will produce more heterozygous offspring – which in cases of widespread overdominance may be preferable [60]. This latter hypothesis predicts that paired individuals are more heterozygous than unpaired ones, and that there is a positive correlation between male and female heterozygosity [61]. To test for non-random mating, the genetic similarity of mated pairs was calculated. The individual genetic distance measure of Kosman & Leonard [62] (D_KL_) was calculated in R within all 108 pairs and was then compared with D_KL_ values within 108 random pairings of the same 54 individuals generated from 10,000 simulations. The vectors of D_KL_ values for the observed and randomised pairings were then compared by the two-sample Kolmogorov-Smirnov (KS) test in R. As a test for significance, the distribution of D from the KS test for 10,000 random comparisons of the simulated data was compared with that for comparisons of the empirical data with each simulacrum. To test if paired worms were more heterozygous than unpaired worms, the Internal Relatedness (IR) [63] was computed for all paired worms and all unpaired worms using GENHET v2.3 [64] in R. The vectors of values for each class were then compared by the Welch two-sample *t*-test in R. The same comparison was also made for males and females separately. Finally, the IRs for the members of each pair were compared by Pearson’s correlation coefficient to detect any correlation between male heterozygosity and that of the associated female.

All plots presented in this study were produced using base functions of R or of ade4 and adegenet. Unless stated otherwise, all statistics were calculated using R and all automation of analysis pipelines was implemented using BASH v4.3.11 [65].

## Results

### Basic population genetic parameters

A total of 274 worms (121 females and 153 males) were recovered from the 10 mice; their distribution among the organs is given in Table 1. It should be noted that loci B and J were excluded from the analysis because both showed heterozygosities differing from expected (under HWE) by > 22.5 % (i.e the threshold of significance at *P* < 0.05, one-tailed test). In addition, LocusJ showed a large heterozygote deficiency F_IS_ 0.2452, whilst LocusB showed a very large heterozygote excess (F_IS_ -0.7689). LocusB also showed a high null allele frequency, estimated to be > −0.2 (approximately -0.3). Finally, IA calculations suggested strong linkage (*r*¯*d* > 0.3) between loci B&J, F&J and H&J; thus strengthening the case for removal of LocusJ. Consequently, the analysis proceeded with seven of the original nine loci. Table 1 also shows that the population structure of the Raw dataset was faithfully recovered by allelematch when assigning each individual to one of 35 clones inferred as present in the sample. Table 1 also reveals a number of alleles that exceeds the number of worms sampled; thus demonstrating the high polymorphism of the microsatellite loci used and their potential in population genetic studies. Of the 263 alleles scored, over 92 % were shared, suggesting considerable levels of gene-flow among the three populations.

**Table 1:** Organotropic distribution of worms and alleles. Counts are given for worms, paired worms, alleles, private (Pr) alleles and total/mean allelic richness, by population, for the alternative datasets used

	Raw Dataset			Filtered Dataset		
	Hpv	L	M	Hpv	L	M
Males	49	26	78	–	–	–
Females	33	13	75	–	–	–
Pairs	42	2	64	–	–	–
Total worms	82	39	153	21	5	9
Alleles	96	76	91	85	39	51
PrAlleles	12	1	7	29	2	3
Richness	85.4/12.2	73.6/10.5	80.3/1.5	44.8/6.4	34.1/4.9	37.3/5.3

*Hpv* Hepatic Portal Vein; *L* Liver; *M* Mesentery; - data unavailable or not applicable

As expected, the values for the Clonal dataset were identical to those for the Raw Dataset

Table 2 shows that no locus was at HWP in the Raw dataset, whilst four loci were at HWP in the Filtered dataset (*P* < 0.005). For the raw dataset most loci showed an excess of heterozygotes (except loci E and F). In contrast, the Filtered dataset showed most loci to be in heterozygote deficit, although this was not significant for LocusH. Despite the failure of individual loci to show HWP, the population overall was in HWP (see K^2^, Table 2). Chi-squared tests, with Yates continuity correction, for HWP by locus within sub-populations, indicated that in the Raw dataset only LocusG in the mesentery sub-population was not significantly out of HWP, whereas for the Filtered dataset no locus differed significantly from HWP (with Bonferroni adjusted α of 0.00238).

**Table 2:** Basic population genetic parameters describing each dataset

	Raw Dataset	Filtered Dataset
F_IS_ by locus	−0.0966 – 0.0571 (A-F)	−0.0957 – 0.0520 (C-E)
Loci with het. def.	**E**, **F**	4 (**EFG**H)
No. loci not in HWP	7 (all at *P <* 0.0002)	4 (DEFG *P* < 0.0024)
K^2^	0.379; *P* = 0.5382	2.667; *P* = 0.1025
Richness by locus	5.723–15.249	3.714–6.941

The range of F_IS_ values is given together with the two loci delimiting the range. Those loci showing a heterozygote deficiency (het. def.) are listed (in bold if significant), all other loci showed excess heterozygosity. K^2^ from Bartlett’s test for total sample (all three populations pooled) wide departure from HWP is given, as is the range of allelic richness across all loci. The values for the Clonal dataset were identical to those for the Raw Dataset

The range of F_IS_ values indicated moderate inbreeding to moderate heterozygote excess for all loci, and the range was similar for both datasets. Table 2 also shows moderate to high levels of allelic richness across loci, the range for the Filtered dataset was of course more narrow. Brookfield’s parametric bootstrap procedure indicated that null allele frequencies at all loci were within the 95 % confidence interval implied by the null distribution, being within −0.05 to 0.05 (for all three datasets).

### Population structure

**Fig. 1:**
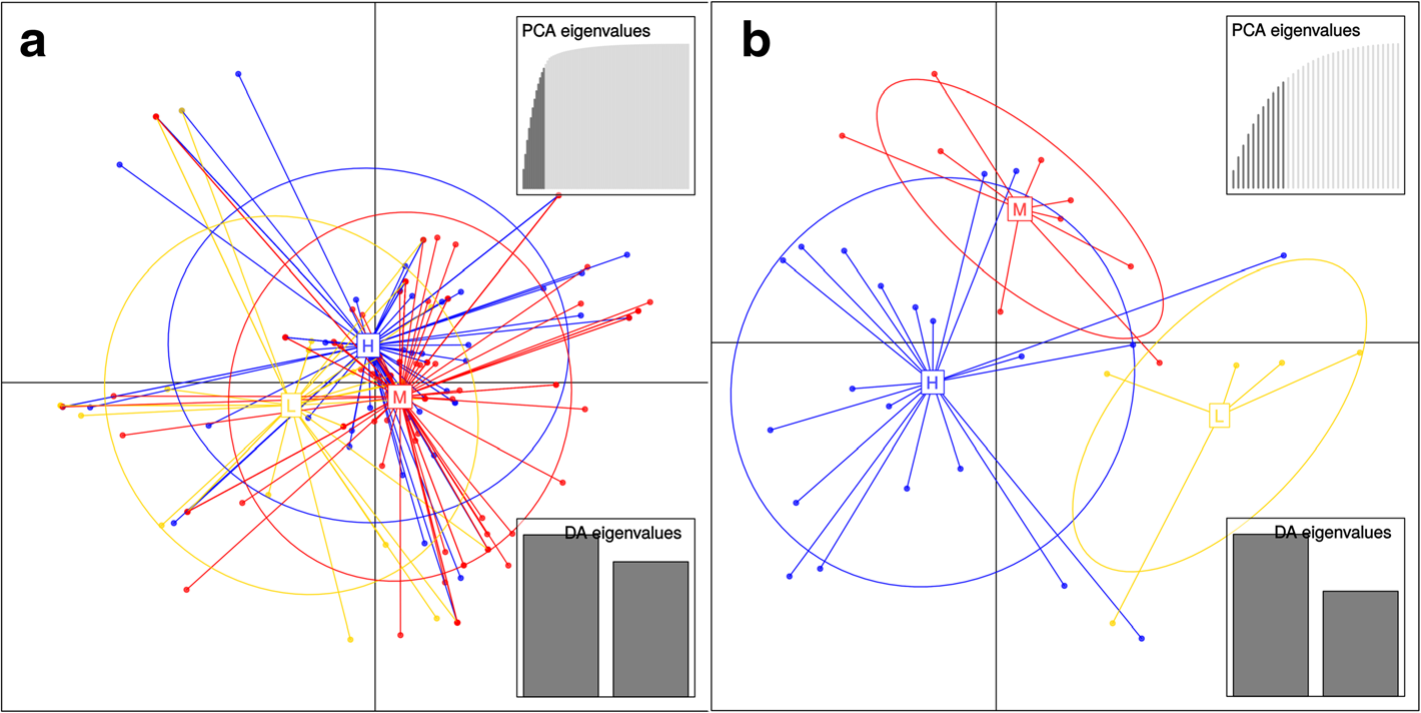
Plot of the linear discriminant analyses for the Raw (**a**) and Filtered (**b**) datasets. A projection of the MLGs, for individuals, is shown onto the plane defined by the axes of the discriminant analyses. Groups are depicted as ellipses showing the variance within the groups, each ellipse being centered about its group mean. H, samples from hepatic-portal-vein; L, samples from liver; M, samples from mesenteric blood vessels. The upper sub-plot shows the eigenvalue screeplot representing the contribution of each axis to the variation, and the lower sub-plot the magnitude of the retained eigenvectors

A discriminant function analysis for population differences in the Raw dataset, using principal components generated from a DUDI PCA revealed some differentiation between the sub-populations sampled from the hepatic-portal-vein (Hpv), liver (L) and mesenteric blood vessels (M) populations, but with some overlap between all three (Fig. 1a). Table 3 shows that Pillai’s test was significant (P < 0.05) for the Raw and Clonal datasets; thus suggesting a significant association between the sampling locations and the principal components. Indeed, in all datasets, over half of the individuals were correctly assigned to their sampling locations, and over 95 % of the mesentery samples were correctly assigned (77 % for the Filtered dataset, see Table 3). The eigenvalues in Table 3 suggest that both principal components explained roughly equal amounts of the variation in the data. As can be seen in Fig. 1b the DAPC for the Filtered dataset suggests a clearer distinction between the sub-populations than inferred for the Raw dataset. As seen in Table 3, the Filtered dataset also showed a better assignment success for the Hpv and L sub-populations. In view of the fact that the DAPC results for the Clonal dataset closely resembled those for the Raw dataset, and that the Filtered dataset might have exaggerated the genetic differentiation among sub-populations, the subsequent studies into population structure related to sex, mating and laboratory host were performed using the Raw dataset only. In addition, pairing relationships were only retained in the Raw dataset.

**Table 3:** Descriptive statistics for the DAPC analyses

	Raw Dataset	Clonal Dataset	Filtered Dataset
Eigenvalues	39.245/36.045	31.165/29.502	10.632/6.885
Pillai’s *P*	0.012	0.010	0.696
BIC	249.662	221.446	42.358
Membership	0.098/0.128/0.954	0.098/0.128/0.954	0.810/0.600/0.778
Prop. Assign.	0.580	0.580	0.771

The eigenvalues are given for DAPC with cross-validation; the significance of the eigenvalues from Pillai’s test (*P* value) is given; the BIC value from the K-means procedure is given for (*K* = 3); also the proportion of successful group membership reassignments is reported by population (Hpv/L/M) and overall (Membership and Prop. Assign., respectively). The membership probabilities below are based on the retained discriminant functions. These statistics are presented for all three datasets

### Potential effects of sex mating and laboratory host

To investigate the effect of sex differences the basic population genetic statistics were re-estimated for each sex individually, the results of this are summarised in Table 4. The Table shows that parameters related to HWE were similar between the sexes and the full dataset. The only notable differences being that all loci showed heterozygote excesses in the females, and that the range of F_IS_ values was greater (than that for the Raw dataset) in the males. Table 4 also shows that in all three cases Pillai’s test was siginificant, the proportions correctly assigned were very similar and the mesenteric sub-population was always the most accurately recovered (possibly because of its smallest variance in the DAPC). PCA plots (Additional file 1: Figure S1) for the males and females were similar to one another and to that for the full dataset. In addition, the retained eigenvalues for the females explained a more even amount of the total variation than did that for the males (Table 4). A Chi-squared test comparing the organotropic distribution of the male and female worms indicated no significant difference between males and females in terms of distribution (Pearson’s Chi-squared test with simulated *P*-value, 5,000 replicates, *Χ*^2^ 3.8291; *P* = 0.1474).

**Table 4:** Summary statistics for single-sex analyses in comparison with those for the Raw dataset

	Females	Males	Raw
F_IS_ by locus	−0.2184 – -0.0349 (G-F)	−0.1264 – 0.0739 (G-F)	−0.0966 – 0.0571 (A-F)
Loci with het. def.	None	D, E, F	E, F
No. loci not in HWP	7 (all at *P <* 0.0002)	7 (all at *P <* 0.0002)	7 (all at *P <* 0.0002)
Eigenvalues	48.976/39.747	8.081/3.051	39.245/36.045
Pillai’s *P*	0.0182	0.043	0.012
Membership	0.061/0.154/0.973	0.163/0.192/0.910	0.098/0.128/0.954
Prop. Assign.	0.636	0.549	0.580

**Fig. 2:**
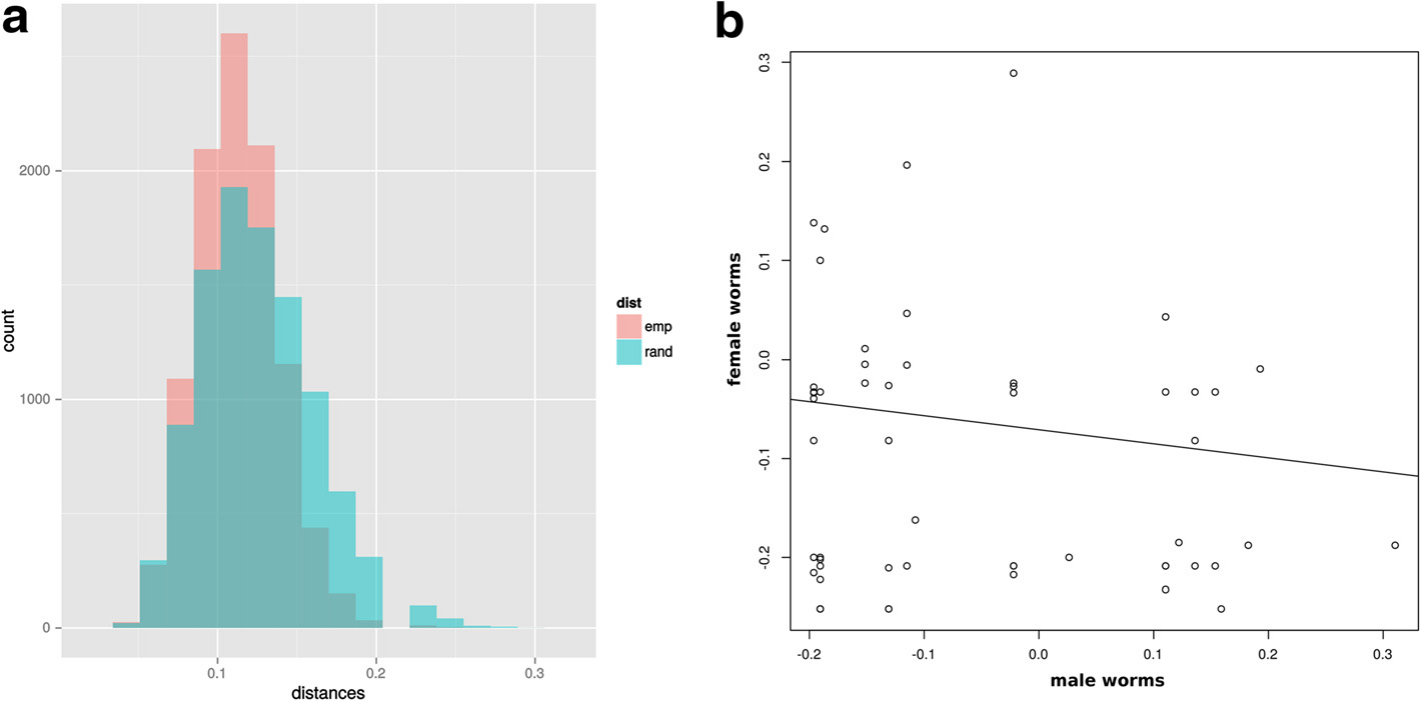
Plots of results of tests for non-random mating. **a**, the distribution of Kolmogorov-Smirnov’s D for observed (emp, pink) and random (rand, blue) pairings of male and female worms. The overlap of the two distributions suggests that there was no significant difference in Kosman and Leonard’s individual genetic distance between members of actual pairings and those of simulated random pairings. **b**, plot of heterozygosities (as Internal Relatedness) and regression line for male and female paired worms

The test for non-random mate choice indicated no significant difference in Kosman and Leonard’s genetic distance within the observed worm pairings compared with that calculated within random pairings of male and female worms. A plot of the empirical and simulated (random pairings) distributions of Kolmogorov-Smirnov’s D is given in Fig. 2a; the two distributions show extensive overlap. Although the paired worms were less heterozygous (−0.0399 versus −0.0557, Welch’s two Sample *t*-test indicated no significant difference between the heterozygosities of paired (mated) worms and worms that were found unpaired (*t* = 0.9280, *P* = 0.3543, two-sided test). Repeating the test for each sex suggested that males in pairs were more heterozygous than unpaired males, but again the difference was not significant (*P* = 0.5055; two-sided test). The same test performed on the female worms indicated that the paired worms were significantly less heterozygous than the worms found as unpaired (*t* = 2.1649; *P* = 0.03262); however, the Bonferroni corrected *P*-value is 0.0979. Next a test was performed for correlation between heterogeneity in males and females within pairs (i.e. for heterozygosity-based assortative mating). The test returned only a weak negative linear correlation between male and female heterozygosity in mating pairs (Pearson correlation coefficient −0.13); the regression line for these data is plotted in Fig. 2b.

**Fig. 3:**
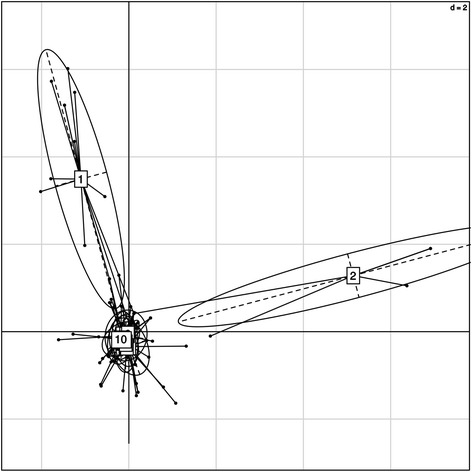
Results of a discriminant analysis with laboratory host set as a factor

Variations among the laboratory hosts used, in terms of the distribution of worms, was also assessed by Chi-squared tests. Pearson’s Chi-squared test (5,000 replicates) indicated a significant difference among the mice in terms of organotropic distribution of worms (*Χ*^2^ = 73.945; *P* = 0.0002). Bonferroni corrected Chi-squared tests on individual mice then revealed a significant bias in mouse 1 (towards L and M), mouse 4 (towards M), mouse 5 (towards M), mouse 9 (towards Hpv and M) and mouse 10 (towards M), *P* < 0.005. Clearly, there was a bias in half of the mice towards worms occupying (or being collected from) the mesenteric blood vessels. Variation in MLGs among infrapopulations in the different mice was assessed using DAPC with host set as a factor. Pillai’s test was significant (*P* = 0.0002) suggesting a significant association between particular host individuals and the principal components, and supporting the findings of the Chi-squared test. Figure 3 depicts the results of the discriminant analysis; in this case projections of the individuals onto the axes, where laboratory host is the factor, indicates that the infrapopulations of worms for mouse 1 and mouse 2 are genetically distinguishable from the worms recovered from the other eight mice.

### Inter- and intra-population variation

The majority of the worms were recovered from the mesenteric blood vessels of the laboratory hosts. Nevertheless, no sub-population was greatly outlying. The mean number of worms per sub-population was 91.3333, with the mesenteric sample (of 153 worms) lying only just outside one Standard Deviation (SD) of the mean (at 149). The other sub-population sizes lay within ± 1 SD. In terms of number of alleles per population, the liver sub-population (with 76 alleles) was just at the 1 SD lower limit of 77 alleles. Regarding number of alleles per locus, the mean was 15, and at the extremes LocusG showed the lowest and LocusH the highest number of alleles. In order to detect any significant differences in number of alleles among loci, *t*-values were calculated for each locus and the Bonferroni corrected, two-tailed (α = 0.05), *P*-values computed; these ranged from 0.7198 to 0.8571. In contrast to the indications of the Chi-squared tests described above, no locus showed heterozygosity differing from that expected by  >17.5 % (the limit of statistical significance at α = 0.05). Together these findings suggested a quite uniform distribution of allelic diversity and conformity to HWP across all loci.

G’_ST_ values with Hedrick’s correction were used as a standardised alternative to F_ST_ appropriate for loci with many alleles. Here G_ST_ is used as an equilibrium estimator of F_ST_ giving an indication of the amount of between sub-population variation relative to within sub-population variation, i.e. an indicator of sub-population differentiation. As underestimation of G’_ST_ may occur with such fast evolving loci as microsatellites, the values for each locus were plotted against their corresponding Jost’s D values. Jost’s D is a measure of the fraction of allelic variation among sub-populations, therefore loci where G’_ST_ is underestimating divergence should also show elevated Jost’s D. Consequently, a plot of G’_ST_ against Jost’s D for each locus, was generated to detect such trends. No such trend was observed (Additional file 2: Figure S2), thus there was no indication of underestimation of divergence by G’_ST_. The standardised G’_ST_ values for among sub-population comparisons were low between Hpv and Liver, and Hpv and Mesentery, suggesting little differentiation between these two sub-populations. The G’_ST_ between the Liver and the Mesentery was an order of magnitude larger, being 0.0254; however, the 95 % confidence interval again included zero, suggesting that much of the variation lay within the overall population, rather than between sub-populations. The G’_ST_ values for all sub-population comparisons are given in Additional file 3: Figure S3 together with their confidence intervals. It should be noted that the difference across all sub-populations was not statistically significant, as indicated by the Chi-squared test comparing organotropic distribution between males and females.

## Discussion

### Heterozygosity

Unlike most earlier studies involving microsatellite variation in *S. japonicum,* which reported heterozygote deficits at all or most loci [9, 10, 12, 13, 15] the present study observed moderate heterozygote excesses across most loci. Only one previous study had reported a heterozygosity excess and this was for miracidia hatched from eggs in feces collected from rodents of villages in hilly areas of Anhui Province, China [11]. The difference in findings might be attributable to the fact that most previous studies omitted individuals with niMLGs, and indeed the present results for the Filtered dataset, which retained only one representative per clone, did indicate a deficit of heterozygotes. Inbreeding, non-random mating or population sub-division (the Wahlund effect) have been suggested as explanations for heterozygote deficits across loci in microsatellite studies of *S. japonicum* [9, 16]. At least one of these effects, the Wahlund effect, can be tested for by simple means, as the magnitude of the Wahlund effect in a mixed sample, as measured by F_IS_, should be proportional to the standardised variance of allele frequency between the two populations in the mixture (i.e. F_ST_) [66]. Consequently, a plot of F_IS_ against F_ST_ (for individual loci) should show a positive linear relationship, with a slope equal to one for equal mixture fractions. In addition, for pairwise tests of loci, it is expected that pairs for which the product of F_ST_ values for the individual loci is largest, will also show the largest *r*^2^ values (where *r* is Hill & Robertson’s *r* [49]) the component of correlation of alleles between the two loci that is attributable to population mixture) [67]. A plot of *r*^2^ against the product of the F_ST_ values should also show proportionality in the presence of a Wahlund effect. In view of this, the dataset with niMLGs removed (the Filtered set) was subjected to these two tests, in order to evaluate this potential cause for the observed heterozygote deficiency. The findings of these tests did not indicate a Wahlund effect as the cause of the observed heterozygote defficiency (see Additional file 4: Figure S4 for plots), with low correlation coefficients for both tests [0.1002 (*P =* 0.8308) and 0.002 (*P* = 0.9942), respectively]. This suggests that in the present study, and perhaps in earlier studies, inbreeding or non-random mating may be responsible for the observed deficiencies in heterozygotes, rather than population structure or age structure (as trickle infections in bovines would facilitate potential mating between older unpaired males and younger females). Inbreeding or non-random mating may be a feature of *S. japonicum* in China as control measures drive down prevalence and populations become fragmented and isolated.

Clonal reproduction, or rapid population expansion from a small number of individuals with many offspring, as might occur during cercariogenesis at the intramolluscan stage of the life-cycle, is expected [68] to generate an excess of heterozygotes relative to random mating. It is therefore possible that the heterozygote deficiency present in *S. japonicum* populations (for example, due to inbreeding) is offset by the excess induced by clonal reproduction at the intramolluscan stage. Should clonal expansion be having an effect on heterozygosity, one would expect to observe an increase in allelic diversity with concomitant decrease in genotypic diversity [69]. Indeed, comparisons of the ratio of allelic richness to genotypic diversity (Shannon-Wiener Index of MLG diversity) for the raw dataset and Filtered dataset (i.e., that adjusted for clonality) did show a significantly larger ratio for the raw dataset (Student’s *t*-test, *t* = 2.6241, *P* = 0.0475); this supports the view that clonality and/or the inclusion of niMLGs has an offsetting effect on a naturally occurring deficiency of heterozygotes in *S. japonicum* populations.

In addition to clonality, an excess of heterozygotes can result from a small effective population size. As already mentioned this scenario might be expected to arise in the face of persistent disease control in China. A common explanation for this is that excesses occur when allele frequencies differ between the sexes in small populations [70]. Indeed, the allele frequencies did differ between the sexes in the present study, as is reflected in the G’_ST_ value between them (0.026) and the difference in extent of genetic variation (depicted by PCA, Additional file 5: Figure S5). Consequently, the heterozygote excess observed in this study could have been the result of small population size. The present study found no evidence for assortative (or disassortative) mating, which could be a cause of heterozygote deficiencies as observed in other studies. It is interesting to note that whilst the males showed moderate (i.e. F_IS_ > 0.1) heterozygote excess at only two loci (and slight excess at two more), the females showed excesses at all loci, with four out of the seven being moderate. Although, according to earlier studies [37], the microsatellite loci were apparently not sex-linked, it is possible that loci in pseudoautosomal regions of sex chromosomes, and which are linked to sex-linked loci, may differ markedly in allele frequencies between the sexes; thus producing an excess of heterozygotes in the heterogametic sex and in the population overall [71]. The pattern difference in excess heterozgosity observed between the sexes in the present study is consistent with such a scenario (the female is the heterogametic sex in *Schistosoma*) spp. but further work to map the loci to the sex chromosomes would be required to support this.

A simpler, and perhaps equally valid, explanation for the excess heterozgosity observed in earlier studies is the small sample sizes upon which all studies of natural populations of *S. japonicum* must be based. Small sample size alone can effect an “excess” heterozygosity proportional to 1/2 N (where N is sample size) [72]. In addition, non-random sampling of kin is quite probable in studies of *S. japonicum*, where samples tend to come from accessible villages, or areas where transmission is known, and sampling is costly and therefore limited. Even where many stool samples are taken or many infected snails collected, these may represent the reproductive output of a small number of adults – a community; this leads to a situation where the small number genotypes, all of which are quite discrete, leads to a slight heterozygote excess in the progeny. Larger samples would lead to mixing of several such communities, thus effecting a counterbalancing “Wahlund effect”. Indeed, a recent study has sought to develop a correction method for such “family structure”, as this was found to lead to heterozygote excess in studies of *Schistosoma mansoni* based on miracidia [73]. Unlike earlier studies, the present study did not aim to compare geographical regions, ecovars or host-groups and so may have inadvertently collected a more random sample than usual, without the heterozygote deficiency caused by including small populations because of their fit to a more complex sampling requirement. It should be noted, however, that the effects of non-random sampling on heterozygosity may be relatively small [74]. Differences in allele frequencies among the sexes as found here can also introduce bias when prevalence is low and single sex infections common, as these will tend to mostly comprise of males this leads to a bias towards MLGs associated with males in areas of low prevalence. Mate choice bias, which was not found in this study, can also lead to organotropic variation that could generate sampling bias, because if a female is paired with a male it is more likely to be found in the mesenteric blood vessels; thus creating a risk that it is more likely to be sampled in a bovine than a small rodent.

### Infrapopulation structure

G’_ST_ values were low between sub-populations, except for the value of 0.0254 between Liver and Mesentery. The lack of differentiation between Hpv and Liver might be attributable to their close proximity and difficulties in determining the precise origin of worms in that anatomical region; however, there was a similar lack of differentiation between Hpv and Mesentery. The differentiation between the Liver and Mesentery sub-populations may be explained by the fact that immature and/or unpaired males tend to predominate there. It could be that differing MLGs between the sexes lie at the root of the difference. The G’_ST_ values for the males and females taken separately lend support to this possibility; these being 0.027 and −0.052, respectively (Hedrick’s G’_ST_ is standardised by division by the maximum theoretical G_ST_ according to the heterozygosity at each locus, hence the negative value). The mating studies indicated that unpaired males were more heterozygous than paired males, although this was not signifincant, it could add to heterozygote excess in samples from small rodents, where the liver and Hpv are often preferentially sampled. Figure 1 (the plot for the DAPC) shows considerable divergence of the three sub-population means, although there was extensive variance leading to overlap among the clusters, the DAPC was significant (for Raw and Clonal datasets). The DAPC again shows that it is the liver sub-population which is differentiated from the other two. Consequently, there may be some genetic differentiation between worms which have matured by week five and have already migrated from the liver to the hepatic-portal-vein and mesenteric blood vessels, in most cases also having paired with a female, and those worms which have not migrated. Such differentiation into fast and slow maturing strains is another source of bias that one might be mindful of in performing future experiments into the transmission ecology of *S. japonicum*.

In addition, there was some evidence for variation between infrapopulations of different laboratory host individuals. The chi-squared test comparing infrapopulations across mice was significant and DAPC indicated that the infrapopulations of two the mice may be genetically distinct populations. In view of this it also appears necessary to use laboratory animals that are as homogeneous as possible and to check patterns of MLGs for such host-induced effects.

## Conclusions

The results have shown that the presence of clones arising from the amplification of parasite numbers during cercariogenesis at the intramolluscan stage, and mutations generating niMLGs at this stage, has a significant impact on heterozygosity, with more loci likely to deviate from HWP. Comparisons of allelic and genotypic diversity between the raw and clone corrected datasets revealed a significant signature of clonal reproduction. Indeed, the effects of clonal expansion appear to be able to offset any deficiency in heterozygotes present in a clone corrected dataset. The presence of clones/niMLGs appeared to have less potential impact on measures of genetic divergence among subpopulations (e.g. F_ST_). Nevertheless, removal of clones/niMLGs did improve the success of assignment of worms to sub-populations by DAPC, and sub-populations appeared more cohesive and distinct in PCAs for datasets with clones removed. There was no evidence that the removal of clones inflated F_ST_ between populations by random loss of MLGs from a sub-sample of the data; however, it is still possible that the reduction in sample size effected departures from HWP. In view of the impact of correcting for clones, it is recommended that studies likely to be affected by this phenomenon should report their results for both corrected and original datasets, as which dataset is best may depend on the aims of the particular study; for example, the relative contribution of clones to a population may be of interest. In addition, compliance with HWP is only critical if subsequent analyses assume HWP.

The study found no evidence that heterozygote deficiencies, found in the clone corrected dataset and typical of earlier studies, were caused by a Wahlund effect. The results did, however, include patterns of heterozygosity differences between the sexes that were consistent with some loci being in pseudoautosomal regions of the sex chromosomes. Mating appeared to be random, with no evidence for assortative or disassortative mating, nor any preference for more heterozygous mates. There was little distinction between the mesentery and hepatic-portal-vein sub-populations; however, the sub-population of the liver formed a relatively discrete sub-population. It is possible that this distinction was, at least in part, due to the predominance of males in the hepatic sub-population, as the males and females showed some genetic differentiation (G’_ST_ 0.024). The effect might be more pronounced if more slowly maturing males represent a distinct strain. The implications of this are that sex-based differences in allele frequencies together with the predominance of males (often immature) in the liver, could lead to biased sampling of worms where infrapopulations of small animals (e.g. rodents) are compared with those of larger animals (e.g. bovines), because the livers and hepatic-portal-veins of small animals are more easily sampled than the mesenteric blood vessels, and the mesentery tends to be the preferred source of worms when sampling larger animals. The present authors have also found that the livers are generally sold as food, with the mesentery being a cheaper and more available source of worms. Consequently, the recommendations of this study are that extreme care is taken to avoid such sampling bias when comparing adult worms between definitive host groups and that results be examined both with and without removal of duplicate MLGs (attributed to clones). Corrections for clones should only be made if tests for the effects of clonal expansion prove positive (e.g. comparisons of allelic and genotypic diversity), and the full dataset used wherever possible. The findings support the case for sampling at the miracidial stage, when studying parasite divergence among host-groups, so as to avoid problems of clonal expansion and niMLGs, possible selection though immune responses of laboratory hosts, as well as sampling bias in collecting worms from hosts. The detection of heterozygote deficiencies probably due to a fragmented and isolated population structure effected by prolonged snail control measures, also calls for a need to employ random and comprehensive sampling strategies for *S. japonicum* in China. Although, the use of miracidia is preferable in the study of host-group specific parasite lineages, it is not suitable for study of transmission ecology and the intramolluscan processes. Additional work is required to develop methods for the study of the entire transmission process for *S. japonicum*.

The present study was limited to a single field sample and to a particular set of microsatellites. The findings may vary with different microsatellite loci or additional samples from the same or other areas, provinces or habitats, and also with other definitive host types as the source of eggs. In addition, the deviations from HWP reflect mating and union of gametes in the previous generation only. Consequently, further work is required to investigate the structure of infrapopulations across generations, regions, habitats and host-groups, and with different microsatellite loci. A true picture of the transmission ecology of *S. japonicum* can only be constructed from the combined results of several population genetic studies covering all of these variables.

## Additional files

Additional file 1: Figure S1. Principal Coordinate Analysis plots. Female (A) and male (B) worms. (PNG 871 kb) Additional file 2: Figure S2. Plot of G_ST_ against Jost’s D for each locus. The points lie roughly on a straight line, with none showing an overly high value of D relative to G_ST_; thus there was no evidence for underestimation of divergence by G_ST_. (PNG 11 kb) Additional file 3: Figure S3. Paiwise G_ST_ values (with Hedrick’s correction) and 95 % confidence intervals for pairwise comparisons among sub-populations. From left to right as plotted; Hpv vs Liver, Hpv vs Mesentery, Liver vs Mesentery. (PNG 79 kb) Additional file 4: Figure S4. Plots of data and regression lines for tests of the Wahlund effect. A. Plot of F_IS_ against F_ST_ (for individual loci); B. Plot of *r* ^2^ against the product of the F_ST_ values for pairs of loci across all sub-populations. Neither plot shows a significant linear relationship, suggesting that a Wahlund effect is less likely for these data. (PNG 208 kb) Additional file 5: Figure S5. PCA depicting genetic diversity among sampled individuals of both sexes. Annotations: 0 females, 1 males. (PNG 47 kb)

## Abbreviations

AMOVA: Analysis of Molecular Variance
DAPC: Discriminant Analysis of Principal Components
DUDI: duality diagram
Hpv: hepatic-portal-vein
HWE: Hardy-Weinberg Equilibrium
HWP: Hardy-Weinberg Proportions
IR: internal relatedness
KS: Kolmogorov-Smirnov (test)
LD: Linkage Disequilibrium
LDA: Linear Discriminant Analysis
MLG: Multilocus Genotype
niMLG: near-identical Multilocus Genotype
PCA: Principal Component Analysis
PcoA: Principal Coordinates Analysis
SD: standard deviation
