# Supplementary figures and images for: An investigation into the potential effects of infrapopulation structure and other sources of sampling error, on population genetic studies of the transmission of Schistosoma japonicum (Trematoda: Digenea)
Source: Parasit Vectors. 2016 Mar 21;9:165. doi: 10.1186/s13071-016-1454-0 (PMC4802887; doi:10.1186/s13071-016-1454-0)

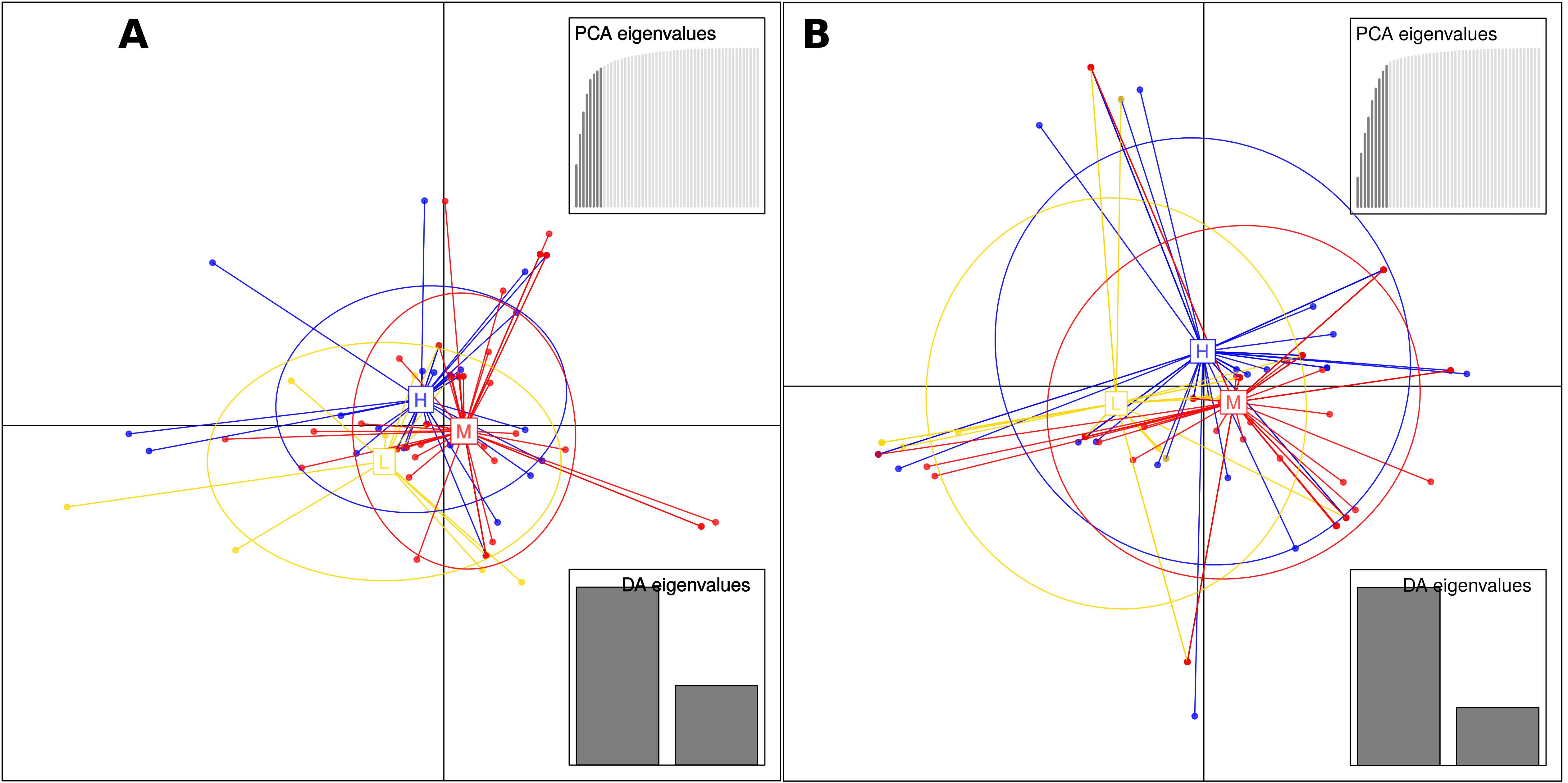

Supplement: Additional file 1: Figure S1. — Principal Coordinate Analysis plots. Female (A) and male (B) worms. (PNG 871 kb) [file 13071_2016_1454_MOESM1_ESM.png]

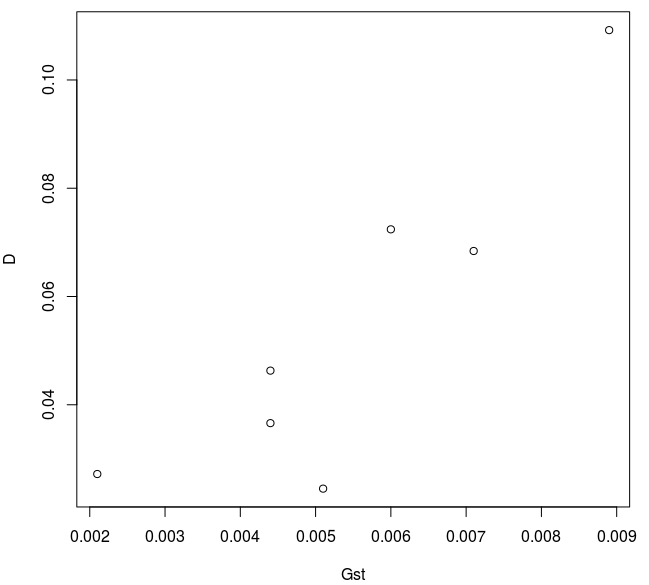

Supplement: Additional file 2: Figure S2. — Plot of GST against Jost’s D for each locus. The points lie roughly on a straight line, with none showing an overly high value of D relative to GST; thus there was no evidence for underestimation of divergence by GST. (PNG 11 kb) [file 13071_2016_1454_MOESM2_ESM.png]

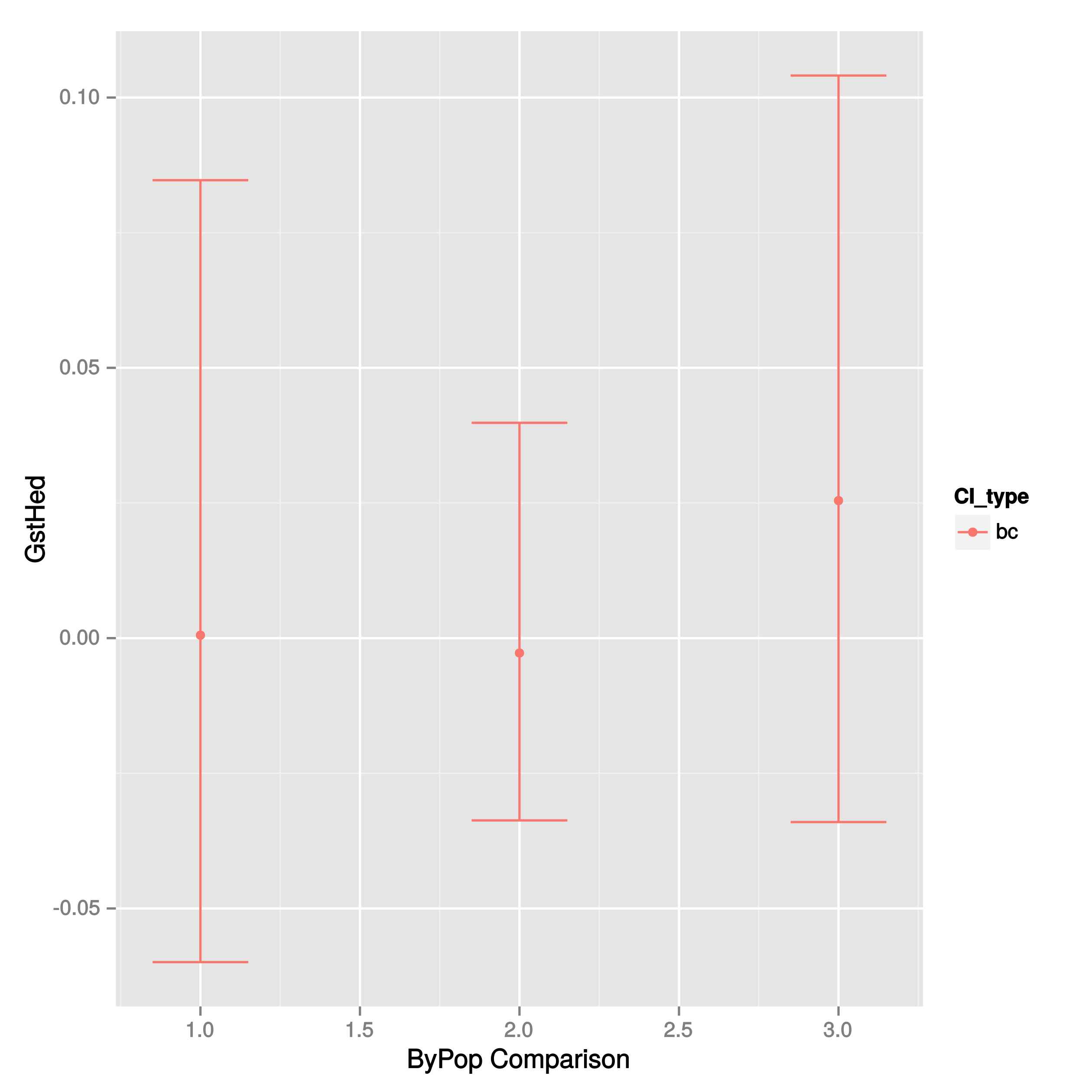

Supplement: Additional file 3: Figure S3. — Paiwise GST values (with Hedrick’s correction) and 95 % confidence intervals for pairwise comparisons among sub-populations. From left to right as plotted; Hpv vs Liver, Hpv vs Mesentery, Liver vs Mesentery. (PNG 79 kb) [file 13071_2016_1454_MOESM3_ESM.png]

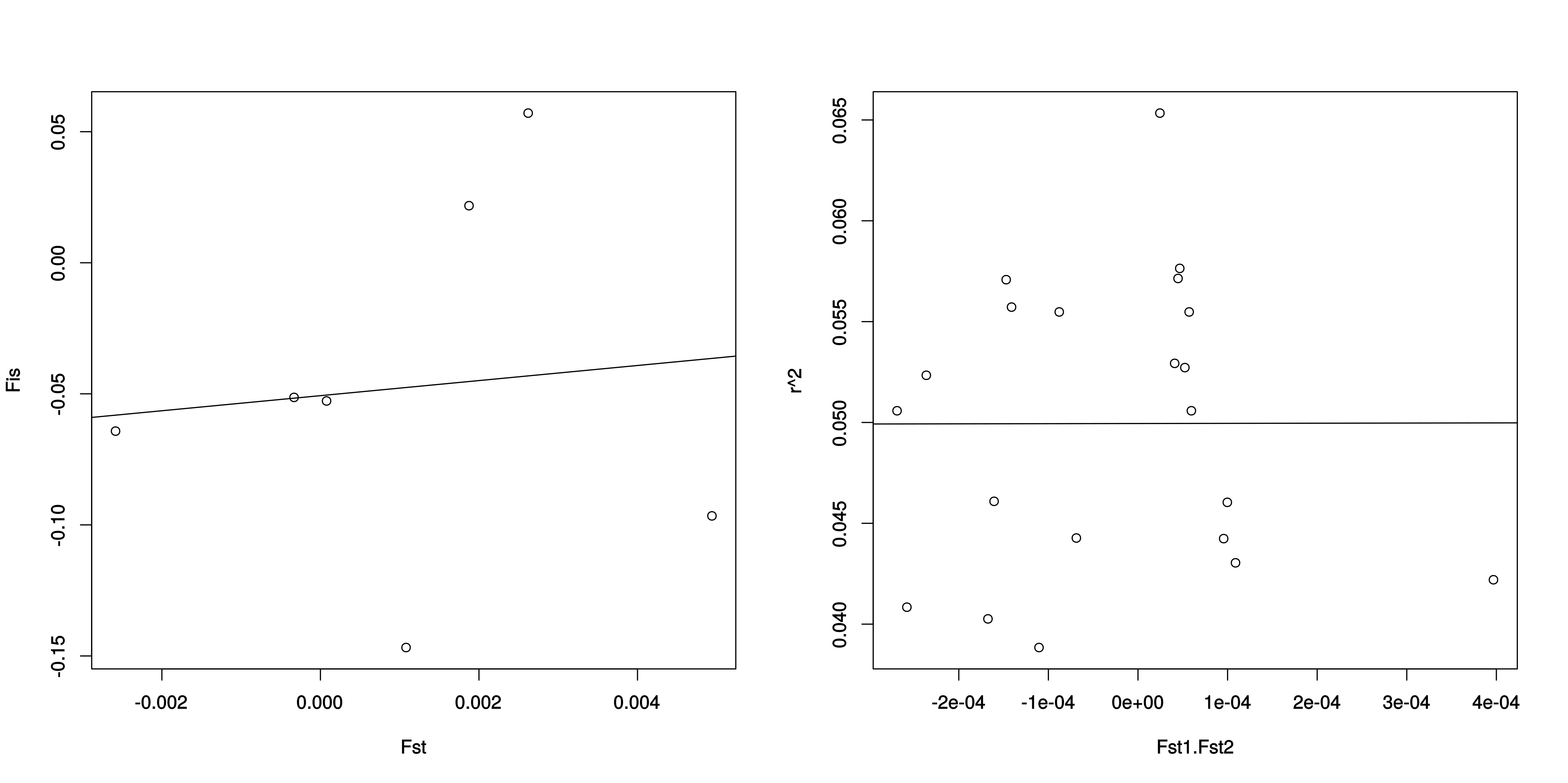

Supplement: Additional file 4: Figure S4. — Plots of data and regression lines for tests of the Wahlund effect. A. Plot of FIS against FST (for individual loci); B. Plot of r 2 against the product of the FST values for pairs of loci across all sub-populations. Neither plot shows a significant linear relationship, suggesting that a Wahlund effect is less likely for these data. (PNG 208 kb) [file 13071_2016_1454_MOESM4_ESM.png]

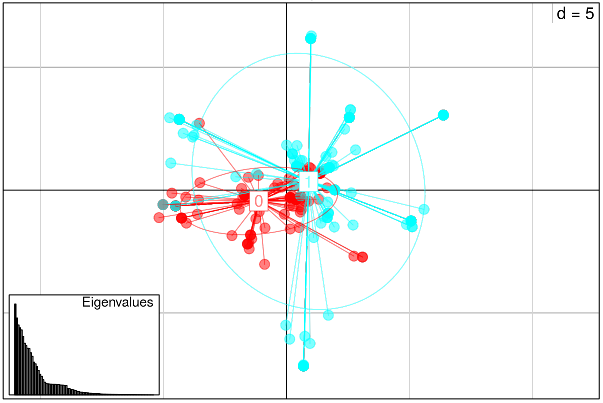

Supplement: Additional file 5: Figure S5. — PCA depicting genetic diversity among sampled individuals of both sexes. Annotations: 0 females, 1 males. (PNG 47 kb) [file 13071_2016_1454_MOESM5_ESM.png]
